# Supplementary material for: HIV-1-envelope trimer transitions from prefusion-closed to CD4-bound-open conformations through an occluded-intermediate state
Source: Comput Struct Biotechnol J. 2024 Nov 12;23:4192–204. doi: 10.1016/j.csbj.2024.11.020 (PMC11617889; doi:10.1016/j.csbj.2024.11.020)
Supplement: Supplementary file 1 — Supplementary material [file mmc1.pdf]

gp120

31 α$_0$

AENLWVTVYYGVPVWKDAETTLFCASDAKAYETEKHNVWATHACVPTDPNPQEIHLENVTEEFNMWKNNM

101 β3 V1V2

VEQMHTDIISLWDQSLKPCVKLTPLCVTLQCTNVTNNITDDMRGELKNCSFNMTTELRDKKQKVYSLFYR

171 β2

LDVVQINENQGNRSNNSNKEYRLINCNTSAITQACPKVSFEPIPIHYCAPAGFAILKCKDKKFNGTGPCP

241 V3

SVSTVQCTHGIKPVVSTQLLLNGSLAEEEVMIRSENITNNAKNILVQFNTPVQINCTRPNNNTRKSIRIG

313

PGQAFYATGDIIGDIRQAHCTVSKATWNETLGKVVKQLRKHFGNNTIIRFANSSGGDLEVTTHSFNCGGE

382 β0 β21

FFYCNTSGLFNSTWISNTSVQGSNSTGSNDSITLPCRIKQIINMWQRIGQAMYAPPIQGVIRCVSNITGL

453

ILTRDGGSTNSTTETFRPGGGDMRDNWRSELYKYKVVKIEPLGVAPTRAKRRVVG

gp41

512 FP HR1$_c$

AVGIGAVFLGFLGAAGSTMGAASMTLTVQARNLLSGIVQQQSNLLRAIEAQQHLLKLTVWGIKQLQARVL

582

AVERYLRDQQLLGIWGCSGKLICTTNVPWNSSWSNRNLSEIWDNMTWLQWDKEISNYTQIIYGLLEESQN

652

QQEKNEQDLLALD

Supplementary Fig. 1: Characteristics in sequence using the HXB2 format.

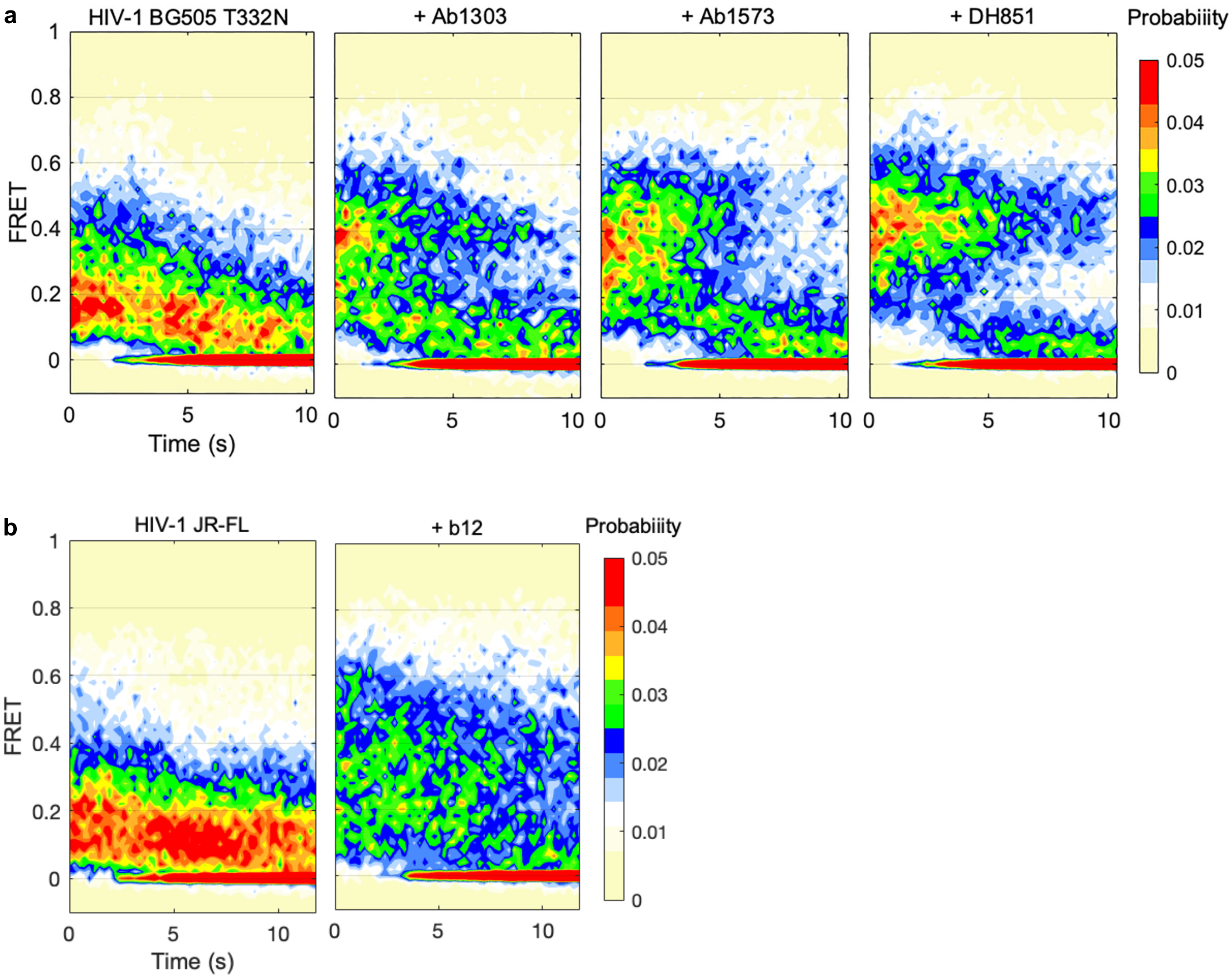

Supplementary Fig. 2: Population contour plots of smFRET trajectories reveal peak shifting of the prevalent conformational state of Env_{BG505} T332N (a) and $Env_{JR\text{-}FL}$ (b) on native virions incubated with occluded intermediate-inducing antibodies.

Population contour plots were compiled of entire smFRET trajectories that dynamic Env trimers on intact virions undergo in the first ten seconds. The contour plots were blindly summed over time (~10 seconds) of dynamic Env trimers included in the corresponding FRET histograms (Figs. 6b – g). Fluorescently labeled Env molecules photobleached within 10 seconds contribute to the 0-FRET (baseline) population as well as baseline fluctuation variations. Population contour plots unbiasedly give overall pictures of conformational ensembles sampled by Env on native virions over time.

a Model fitting involves CBO (~0.3 FRET), corresponding to indicated histograms in Fig.6

Conformational populations of Ab-incubated Env on native virions	Curve fitting R^2	RMSE	PT State	PC State	CBO State
			µ: 0.12 ± 0.02	µ: 0.65 ± 0.04	µ: 0.30 ± 0.01
			σ: 0.08 ± 0.01	σ: 0.14 ± 0.01	σ: 0.11 ± 0.01
Env_{BG505}	0.9934	6.504e-4	46% ± 7%	20% ± 10%	34% ± 10%
Env_{BG505} + Ab1303	0.9543	0.0013	21% ± 11%	37% ± 16%	42%± 19%
Env_{BG505} + Ab1573	0.9710	9.729e-4	18% ± 11%	38% ± 19%	44% ± 22%
Env_{BG505} + DH851	0.9218	0.0017	13% ± 10%	45% ±18%	42% ± ULM%
Env_{JR-FL}	0.9937	7.131e-4	55% ± 9%	15% ± 6%	30% ± 11%
Env_{JR-FL} + b12	0.9696	0.0011	19% ± 8%	33% ± 18%	48% ± 19%

b Model fitting involves OI (~0.4 FRET)

Conformational populations of Ab-incubated Env on native virions	Curve fitting R^2	RMSE	PT State	PC State	OI State
			µ: 0.12 ± 0.02	µ: 0.65 ± 0.04	µ: 0.4 ± 0.01
			σ: 0.08 ± 0.01	σ: 0.14 ± 0.01	σ: 0.11 ± 0.01
Env_{BG505}	0.9705	0.0013	54% ± 5%	11% ± ULM%	35% ± 8%
Env_{BG505} + Ab1303	0.9904	6.138e-4	33% ± 4%	24% ± 8%	43% ± 9%
Env_{BG505} + Ab1573	0.9813	8.082e-4	30% ± 8%	24% ± 9%	46% ± 9%
Env_{BG505} + DH851	0.9932	4.903e-4	24% ± 5%	27% ± 9%	49% ± 9%
Env_{JR-FL}	0.9669	0.0016	62% ± 7%	8% ± 8%	30% ± 12%
Env_{JR-FL} + b12	0.9892	6.460e-4	31% ± 8%	22% ± 12%	47% ± 12%

c Model fitting involves both CBO (~0.3 FRET) and OI (~0.4 FRET) – overfitting and uncertainty

Conformational populations of Ab-incubated Env on native virions	Curve fitting R^2	RMSE	PT State	PC State	CBO State	OI State
			µ: 0.12 ± 0.02	µ: 0.65 ± 0.04	µ: 0.3 ± 0.01	µ: 0.4 ± 0.01
			σ: 0.08 ± 0.01	σ: 0.14 ± 0.01	σ: 0.06 ± 0.01	σ: 0.06 ± 0.01
Env_{BG505}	0.9967	4.667e-4	50% ± 7%	18% ± 9%	18% ± 15%	14% ± 17%
Env_{BG505} + Ab1303	0.9867	7.182e-4	35% ± ULM%	31% ± ULM%	8% ± ULM%	26% ± ULM%
Env_{BG505} + Ab1573	0.9717	9.917e-4	28% ± ULM%	34% ± ULM%	16% ± ULM%	22% ± ULM%
Env_{BG505} + DH851	0.9833	7.875e-4	23% ± ULM%	38% ± ULM%	13% ± ULM%	26% ± ULM%
Env_{JR-FL}	0.9924	7.933e-4	59% ± 5%	16% ± 12%	12% ± 17%	13% ± 13%
Env_{JR-FL} + b12	0.9824	8.356e-4	30% ± 6%	29% ± 13%	15% ± 15%	26% ± 20%

*ULM indicates 'uncertainty larger than mean'

d

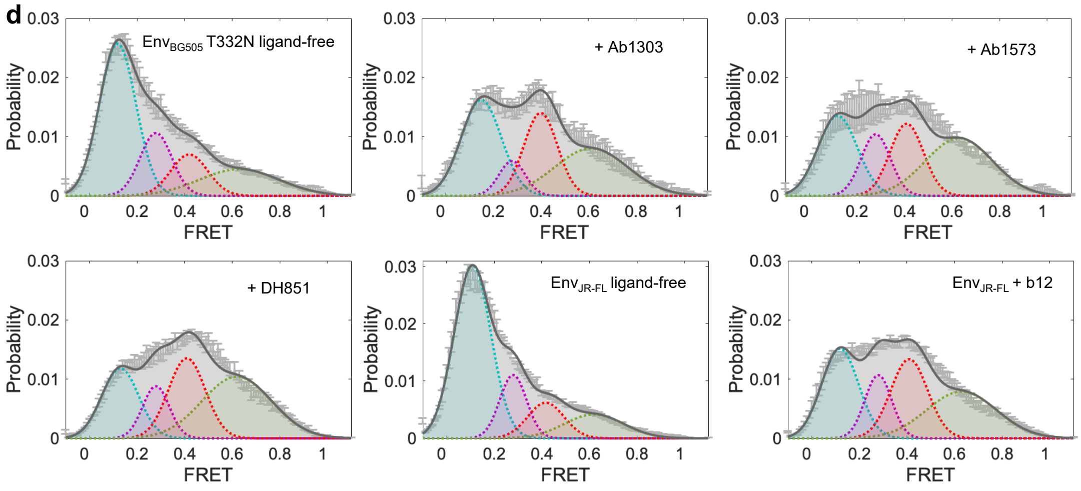

Supplementary Fig. 3: Comparison of goodness of model fitting of FRET histograms with three and four states and fitting parameters.

a, b Constrained model-fitting of FRET histograms of Ab-incubated Env on native virions into a sum of three-state Gaussian/Normal $N(\mu, \sigma^2)$ distributions that includes CBO instead of OI worsens the goodness of fitting. Parameters (μ, σ) chosen for fitting were determined based on visual inspection of all trajectories that exhibit state-to-state transitions and the idealization of individual trajectories using multi-state Hidden Markov modeling. The probability of each state Env occupies was presented as mean ± s.e.m (uncertainty). ULM (s.e.m) comes from unfavorable fitting (with less than 95% confidence). R^2 and RMSE (Root Mean Square Deviation) evaluate the goodness of fitting. A value of R^2 closer to 1 and/or RMSE closer to 0 indicates a better fit/prediction. Cyan-colored R^2 and RMSE indicate worse fitting/prediction. Parallel comparisons of R^2, RMSE, and probability s.e.m obtained from model fitting involving CBO and OI, imply a better presentation of the conformational population of Ab-incubated Env using OI state than CBO state.

c, d Statistics and associated curve fitting of FRET histograms with a sum of four Gaussian distributions. Fitting to 4-state instead of 3-state increases the likelihood of overfitting and causes an increase in uncertainty, as implied by the elevated uncertainty of state occupancy or even ULM (generated with less than 95% confidence bounds). Uncertainty close to the level of mean was highlighted.

a

Antibody-stabilized conformational states of Env on native virions	Anti-Env antibodies or ligands							
Pre-trigged (PT)	10-1074[1]	2G12[2]	3BNC117[1]	PG9[2]	PG16[2]	PGT122[2]	Temsavir[1,2,3]	VRC01[2]
CD4-bound open (CBO)	CD4 mimetics[2]	eCD4-Ig[1]	F105[1]	sCD4$_{D1D2}$-Igαtp[1,3,4]	sCD4+17b[1,4]			
Prefusion closed (PC)	Cow-1[1]	Cow-8[1]	Cow-9[1]	Cow-10[1]	PGT151[1]	VRC34[5]		

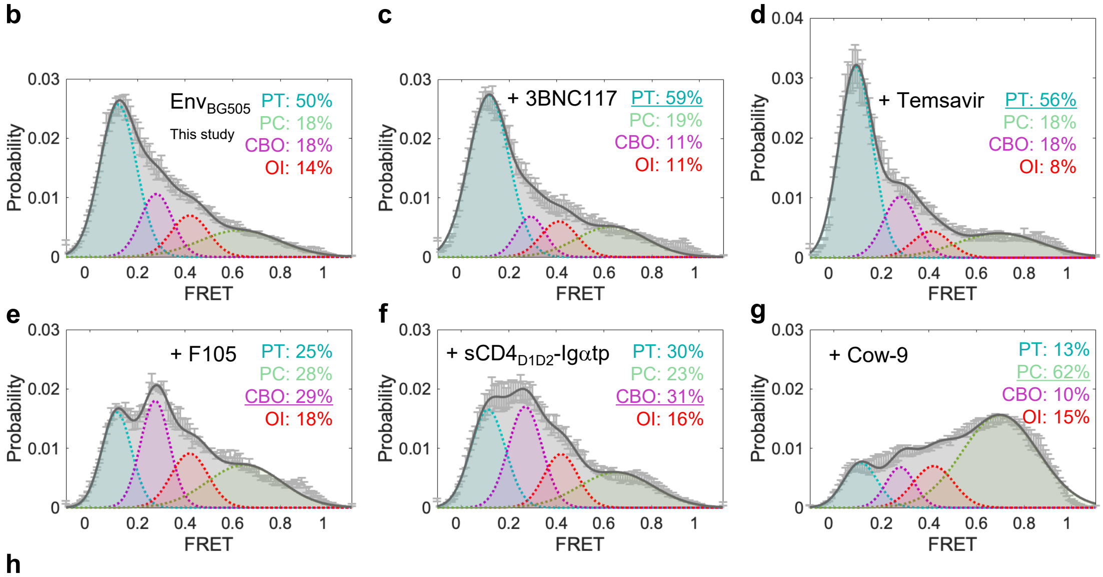

h

Conformational populations of Ab-incubated Env on native virions	Curve fitting R^2	RMSE	PT State	PC State	CBO State	OI State
			μ: 0.12 ± 0.02	μ: 0.65 ± 0.04	μ: 0.3 ± 0.01	μ: 0.4 ± 0.01
			σ: 0.08 ± 0.01	σ: 0.14 ± 0.01	σ: 0.06 ± 0.01	σ: 0.06 ± 0.01
Env$_{BG505}$ (data in this study)	0.9967	4.667e-4	50% ± 7%	18% ± 9%	18% ± 15%	14% ± 17%
Env$_{BG505}$ + 3BNC117	0.9915	7.481e-4	59% ± 6%	19% ± 11%	11% ± 25%	11% ± 30%
Env$_{BG505}$ + Temsavir	0.9891	9.352e-4	56% ± 3%	18% ± 5%	18% ± ULM%	8% ± ULM%
Env$_{BG505}$ + F105	0.9852	7.777e-4	25% ± 4%	28% ± ULM%	29% ± ULM%	18% ± 4%
Env$_{BG505}$ + sCD4$_{D1D2}$-Igαtp	0.9951	4.810e-4	30% ± 2%	23% ± 3%	31% ± ULM%	16% ± ULM%
Env$_{BG505}$ + Cow-9	0.9814	6.821e-4	13% ± 4%	62% ± 7%	10% ± 12%	15% ± 15%

*ULM indicates 'uncertainty larger than mean'

i

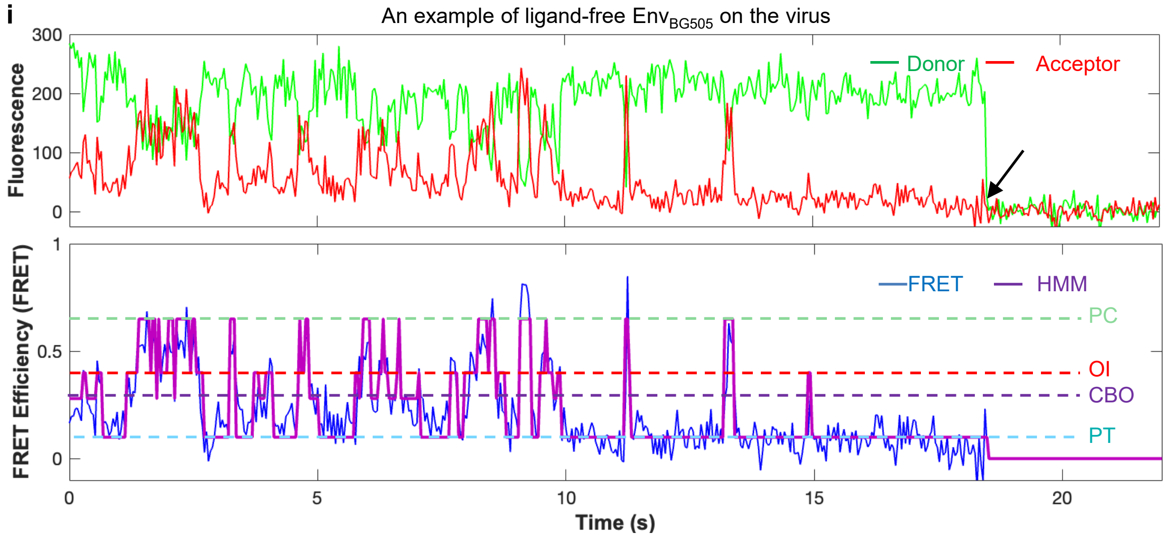

Supplementary Fig. 4: Refitting of prior smFRET datasets with tested antibodies by 4-state Gaussian model indicates no population enrichment in the OI state.

a Table listing specific stabilized states of Env by individual antibodies in previous studies (refs. 1-5).

b – g Example histograms describing enhancement of a prior state occupied by the ligand-free Env (**b**) by an antibody/ligand in each class, including PT-stabilizing (**c, d**), CBO-stabilizing (**e, f**) and PC-stabilizing (**g**) ligands. Histogram in b is the same as Fig.6b, whereas histograms in c – g are from published work (ref. 1). The occupancy of dominant state is underlined.

h Statistics and associated curve fitting of FRET histograms (**b – g**) with a sum of four Gaussian distributions, as described in Supplementary Information.

i Example fluorescence (donor, green; acceptor, red) trajectory and resulting FRET efficiency trajectory (FRET efficiency, blue; hidden Markov modeling – HMM idealization, magenta) of a dually labeled Env$_{BG505}$ on an intact virion. The black arrow points to the single-step photobleaching. Four FRET-populated states are indicated as color-coded dashed lines.

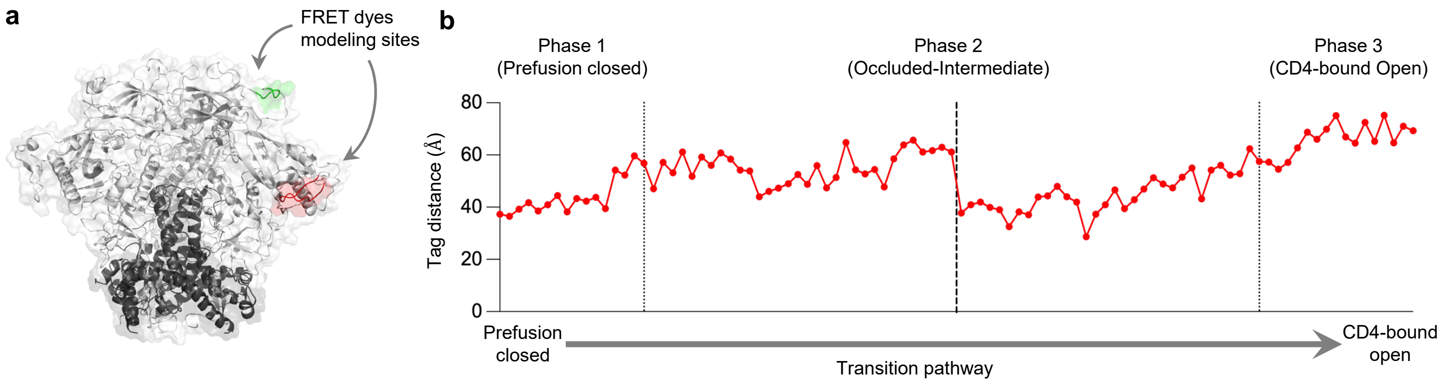

c FRET and tag distance of Phase 1, 2, and 3

Phase	1	2	3
FRET efficiency	0.65	0.4	0.3
Tag distance (Å)	37.3	28.6 – 65.7 (Average 50.2)	69.2

Supplementary Fig. 5: The correlation between tag distance and smFRET.

a Schematic figure of modeled fluorescent donor-acceptor labels on an Env_{BG505} trimer.

b Tag distance over the transition pathway.

c FRET and tag distance at each phase. The tag distances at phase 1 and 3 are from the farthest end. For phase 2, minimum to maximum value along with average distance are shown.

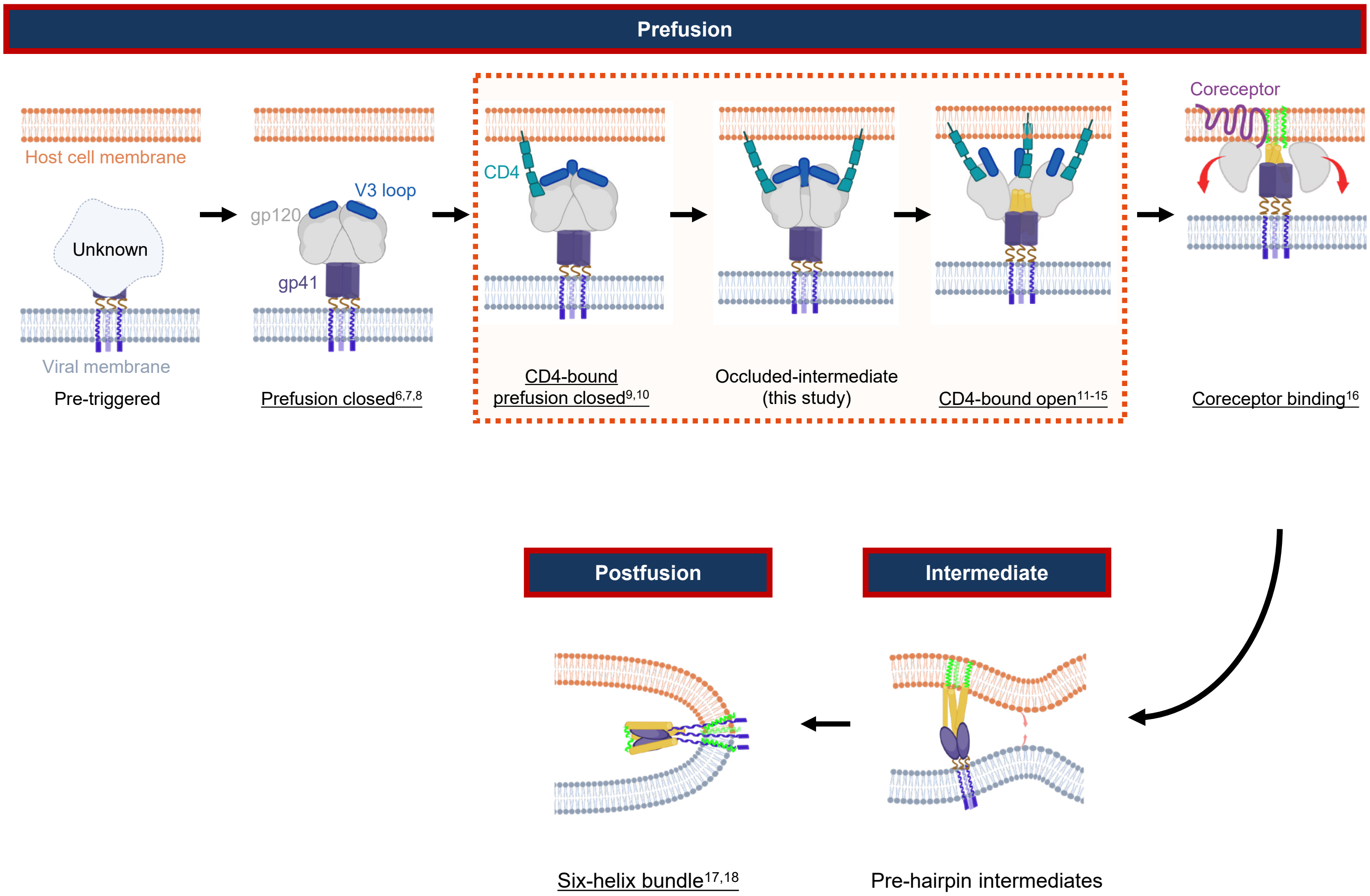

Supplementary Fig. 6: Schematic figure depicting the scope of current study in orange box over HIV-1 Env fusion process.
Note that for logistical reasons, dynamics were performed with three CD4s throughout the trajectory. Conformations with underline labels have determined residue-level structures.

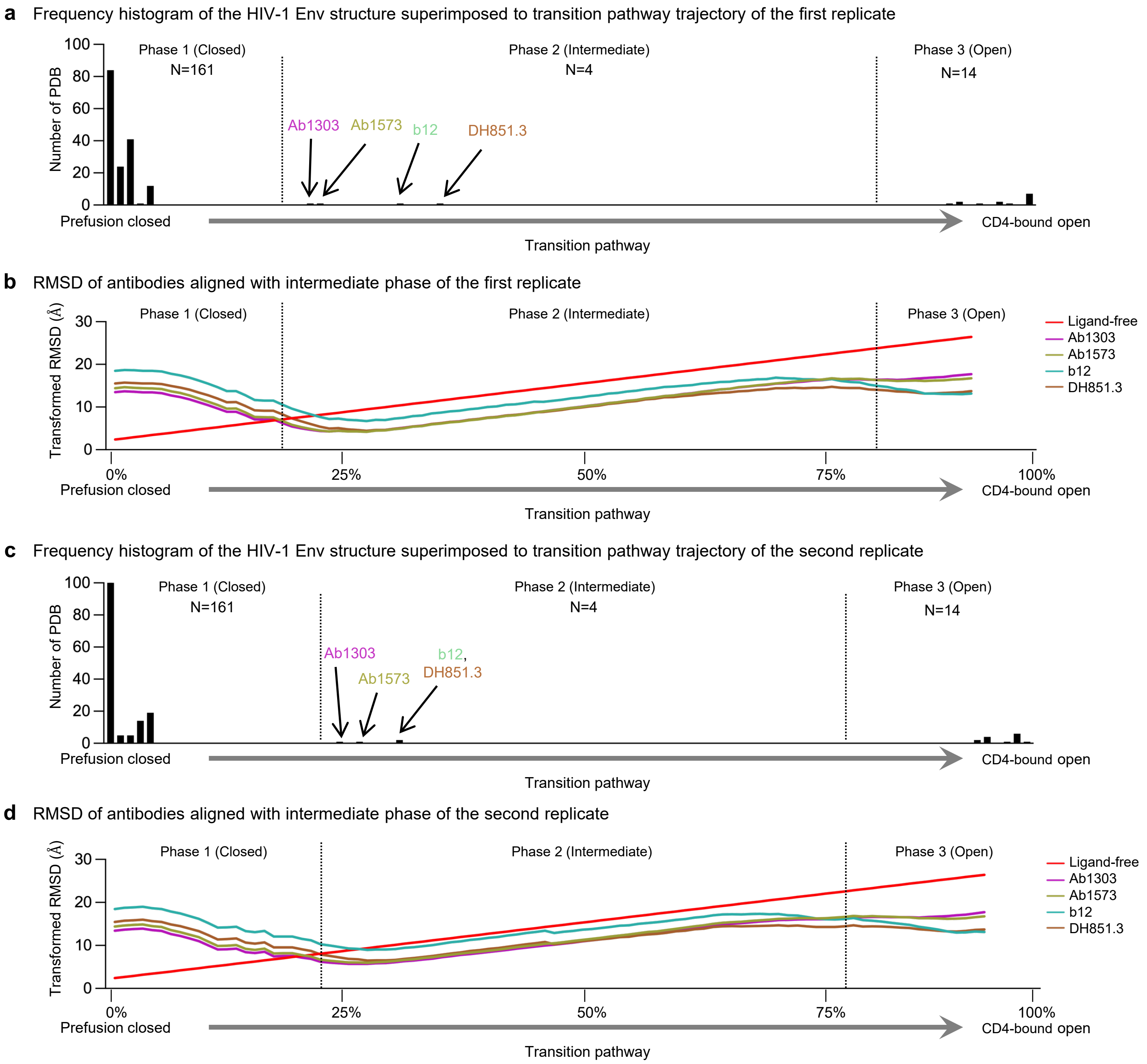

Supplementary Fig. 7: Two additional simulated replicates show similar results, with the majority of the trajectory demonstrating occluded intermediate conformations.

a, c Histograms of the first and second replicates display the number of PDB structures (N = 179) of HIV-1 Env trimers aligned with the transition pathway trajectory. Four antibodies distinctly induce occluded intermediate conformations.

b, d The transformed RMSDs of four antibodies that were identified as inducers of the occluded intermediate state of the first and second replicate.

Supplementary References

1. Lu ML*, et al.* Associating HIV-1 envelope glycoprotein structures with states on the virus observed by smFRET. *Nature* **568**, 415-+ (2019).
2. Munro JB*, et al.* Conformational dynamics of single HIV-1 envelope trimers on the surface of native virions. *Science* **346**, 759-763 (2014).
3. Zou, *et al.* Long-Acting BMS-378806 Analogues Stabilize the State-1 Conformation of the Human Immunodeficiency Virus Type 1 Envelope Glycoproteins. *J Virol* **94**, e00148-20 (2020).
4. Ma XC*, et al.* HIV-1 Env trimer opens through an asymmetric intermediate in which individual protomers adopt distinct conformations. *Elife* **7**, e34271 (2018).
5. Kong, *et al.* Fusion peptide of HIV-1 as a site of vulnerability to neutralizing antibody *Science* **352**, 828-833 (2016).
6. Lyumkis D*, et al.* Cryo-EM structure of a fully glycosylated soluble cleaved HIV-1 envelope trimer. *Science* **342**, 1484-1490 (2013).
7. Julien JP*, et al.* Crystal structure of a soluble cleaved HIV-1 envelope trimer. *Science* **342**, 1477-1483 (2013).
8. Pancera M*, et al.* Structure and immune recognition of trimeric pre-fusion HIV-1 Env. *Nature* **514**, 455-461 (2014).
9. Liu, *et al.* Quaternary contact in the initial interaction of CD4 with the HIV-1 envelope trimer. *Nat Struct Mol Biol* **24,** 370-378 (2017).
10. Dam KA, Fan C, Yang Z, Bjorkman PJ. Intermediate conformations of CD4-bound HIV-1 Env heterotrimers. *Nature* **623**, 1017-1025 (2023).
11. Ozorowski G*, et al.* Open and closed structures reveal allostery and pliability in the HIV-1 envelope spike. *Nature* **547**, 360-+ (2017).
12. Wang H, Cohen AA, Galimidi RP, Gristick HB, Jensen GJ, Bjorkman PJ. Cryo-EM structure of a CD4-bound open HIV-1 envelope trimer reveals structural rearrangements of the gp120 V1V2 loop. *Proc Natl Acad Sci U S A* **113**, E7151-E7158 (2016).
13. Yang Z, Wang H, Liu AZ, Gristick HB, Bjorkman PJ. Asymmetric opening of HIV-1 Env bound to CD4 and a coreceptor-mimicking antibody. *Nat Struct Mol Biol* **26**, 1167-1175 (2019).
14. Jette CA, Barnes CO, Kirk SM, Melillo B, Smith AB, 3rd, Bjorkman PJ. Cryo-EM structures of HIV-1 trimer bound to CD4-mimetics BNM-III-170 and M48U1 adopt a CD4-bound open conformation. *Nat Commun* **12**, 1950 (2021).
15. Wang H, Barnes CO, Yang Z, Nussenzweig MC, Bjorkman PJ. Partially Open HIV-1 Envelope Structures Exhibit Conformational Changes Relevant for Coreceptor Binding and Fusion. *Cell Host Microbe* **24**, 579-592 e574 (2018).
16. Shaik MM, *et al.* Structural basis of coreceptor recognition by HIV-1 envelope spike. *Nature* **565**, 318-323 (2018).
17. Chan DC, et al. *Cell* **89**, 263-273 (1997).
18. Weissenhorn W, *et al.* Atomic structure of the ectodomain from HIV-1 gp41. *Nature* **387**, 426–430 (1997).
